# Effects of Exogenous Supplementation of Progesterone on Conception Rates in Lactating Murrah Buffaloes

**DOI:** 10.3390/vetsci12030261

**Published:** 2025-03-11

**Authors:** Rajesh Kumar, Jagat Bir Phogat, Rakesh Kumar Sharma, Sushil Kumar Phulia, Jerome Andonissamy

**Affiliations:** 1 Animal Physiology and Reproduction Division, ICAR—Central Institute for Research on Buffaloes, Hisar 125001, Haryana, India; rajesh.kumar2@icar.gov.in (R.K.); sushil.phulia@icar.gov.in (S.K.P.); 2 Department of Veterinary Gynaecology and Obstetrics, Lala Lajpat Rai University of Veterinary and Animal Sciences, Hisar 125001, Haryana, India; phogat_jagat@rediffmail.com

**Keywords:** buffalo, Murrah, progesterone, ovulation, conception rate

## Abstract

The objective of this study was to deduce the effect of exogenous supplementation of progesterone as an intramuscular injection at the time of artificial insemination (AI) on time of ovulation and conception rate in lactating Murrah buffaloes. A total of 30 buffaloes were included in the experiment and randomly divided into two groups (Treatment, *n* = 13 and Control, *n* = 17). Only those buffaloes which were in heat according to visual observation and had clear vaginal discharge, good uterine tone and a large follicle (>12 mm on ultrasound scanning) were reported. Ultrasound scanning was carried out at 6 h intervals after insemination until ovulation. The results revealed that significantly higher numbers of buffaloes ovulated within 24 h post AI in the control group (82.4%) as compared to only 15.4% in the treatment group. In the treatment group, 53.8% of ovulations occurred after 24 h post AI, whereas in the control group only 11.8% of ovulations occurred after 24 h post AI. Up to 96 h post AI, 30.8% of buffaloes in the treatment group and only 5.9% of buffaloes in the control group remained anovulatory. No significant difference was found in CL size between the treatment (226.5 ± 17.4 mm^2^) and control (238.9 ± 7.9 mm^2^) groups. Following insemination, 52.9% of buffaloes in the control group conceived, whereas in the treatment group, only 38.5% of buffaloes conceived.

## Simple Summary

This study was carried out to deduce the effect of exogenous supplementation of progesterone as an intramuscular injection at the time of artificial insemination on time of ovulation and conception rate in lactating Murrah buffaloes. Study buffaloes were included in the experiment and randomly divided into two groups and those buffaloes which were in heat according were reported. This study revealed that less numbers of buffaloes ovulated following progesterone treatement and also more treated animals ovulated post artifical insemaintion. The study found that no difference was found in corpus luteum size between the groups and lower number of treated animals conceived. Hence, the use of exogenous progesterone at the time of artificial insemination has no and/or negative effects on the fertility in buffaloes.

## 1. Introduction

Progestogens are a group of hormones with similar physiological activities, the most important of which is progesterone (P_4_), which plays a key role in the regulation of the estrous cycle. During the estrous cycle, peripheral P_4_ concentrations were found to be similar in buffaloes and cattle [1,2,3]. The concentration of P_4_ is minimal on the first day of the estrus cycle (0.1 ng/mL), rising to peak concentrations of 1.6–3.6 ng/mL on days 13 to 17, before declining to basal levels at the onset of the next estrus cycle [4]. It has been found that inadequate luteolysis can result in an elevation in circulating progesterone and a reduction in fertility after artificial insemination (AI). This is clearly a problem with some animals during timed AI programs [5,6] and may also be a problem in AI programs based on the detection of estrus. In 5–30% of cows, complete regression of the corpus luteum (CL) did not occur following prostaglandin treatment administered according to the Ovsynch protocol [7,8,9]. Minor elevation in P_4_ concentration shortly after AI has a detrimental effect on fertility [10,11]. P_4_ is an effective blocker of tonic secretion and LH surge release in bovines, as P_4_ alters sperm or oocyte transport by altering uterine or oviductal contractility and reducing embryo development by reducing endometrial thickness.

It has been reported that P_4_ administration during the early luteal phase should cause shortening of the dominant phase of wave 1 in two-wave patterns, and will result in the early emergence of the second wave and the need for the third wave to emerge before luteal regression occurs, thereby transforming the two-wave pattern into a three-wave pattern [12]. Therefore, exogenous P_4_ administration during the early inter-ovulatory interval can suppress the growth of the dominant follicle (DF) of wave 1, resulting in the early emergence of wave 2. By administration of decreasing doses of P_4_ (150, 100, 75, 50 and 25 mg) into buffalo heifers, starting from day 0 (day of ovulation) and continuing to day 5 of the estrus cycle [13]. P_4_ treatment significantly increased the proportion of 3-wave cycles as compared to controls (75% vs. 30%). However, there were no significant differences in the mean diameters of the ovulatory follicles or CLs between the heifers in the two groups. The pregnancy rates of treated heifers (43.8%) and control heifers (40.0%) did not differ significantly. P_4_ is administered via the intra-vaginal route by means of intra-vaginal devices. Initially, sponges were used, which posed the problem of retention. This led to the development of silastic coils [14] and finally to PRID and CIDR, which came into existence to provide better retention properties and also to release progesterone at a controlled rate.

The incorporation of estradiol benzoate (EB) as a luteolytic agent has enabled short-term PRID/CIDR treatments to synchronize estrus effectively. It has been studied that the effect of fixed-time artificial insemination in Murrah buffaloes after synchronizing their estrus cycles using CIDR/EB and obtained a conception rate of only 22.8% [15]. It has been observed better estrus and conception rates in buffaloes when prostaglandin was administered on the day of CIDR removal than for those treated with CIDR alone [16]. The conception rate was 30.0% in the CIDR group under farm conditions during the low breeding season. The conception rates did not differ between Ovsynch (70.0%) and CIDR (54.5%) groups under farm conditions in peak breeding season. Considering these findings, the present study has been designed to study the effect of exogenous supplementation of progesterone at the time of AI on conception rates in lactating Murrah buffaloes in an organized herd.

## 2. Materials and Methods

### 2.1. Place of Work and Study Animals

The research work was carried out in the animal farm section of the ICAR-Central Institute for Research on Buffaloes, Hisar (29.18° N latitude and 75.70° E longitude). The farm has a herd of nearly 550 animals of the Murrah breed that are maintained under a semi-intensive system of management. The study was conducted from September 2019 to August 2021. All procedures were conducted with the approval of the Institutional Animal Ethics Committee. A total of 30 buffaloes were included in the experiment and randomly divided into two groups (Treatment, *n* = 13 and Control, *n* = 17). All experimental buffaloes followed a standard lactation period of 305 days. Only those buffaloes that were reported on heat by visual observations and had clear vaginal discharge, good uterine tone and a large follicle (>12 mm) on ultrasound scanning were included in the group. In the present experiment, the time has been calculated with respect to mid heat and insemination. Buffaloes reported to be on heat in the morning were examined rectally in the evening and inseminated, while those reported in the evening were examined and inseminated using a single straw the next morning. Ultrasound scanning was carried out at 6 h intervals without holding the ovary, using the size of follicles and the sudden disappearance of the largest ovulatory follicle as indications of ovulation.

### 2.2. Study Groups

The buffaloes in the treatment group (*n* = 13) received an intramuscular injection (750 mg) of hydroxyprogesterone caproate (Duraprogen, 3 mL, Vetcare) at the time of AI, whereas those in the control group (*n* = 17) received no treatment. All buffaloes were inseminated mid-estrus, as defined earlier.

### 2.3. Blood Sampling

Five ml of blood was collected from the jugular vein in heparinized 10 mL vacutainer vials at the time of AI and on days 5 and 12 post AI. Blood was brought to the laboratory on a cool cage soon after collection. Centrifugation was conducted at 250× *g* for 15 min to separate plasma, and plasma aliquots were stored at −80 °C until analyzed for progesterone levels.

### 2.4. Estimation of P_4_

The concentrations of P_4_ were measured in plasma samples from 30 buffaloes collected using a P_4_ EIA kit, assessed using a solid-phase enzyme immunoassay (XEMA, Kyiv, Ukraine) and expressed as ng/mL (mean ± SE). The procedure described by the manufacturer was used and a standard curve was prepared using known standards. The coefficients of intra-assay and inter-assay variation were <8% and <10%, respectively. The sensitivity of the kit was 0.0786 ng/mL.

### 2.5. Ultrasonography

Transrectal ultrasonography was performed using a Hitachi Aloka Ultrasound (Tokyo, Japan) machine, which showed the overt signs of estrus for confirmation of true estrus and measurement of the size of dominant follicles at mid-estrus just before AI. Ultrasound was continued at 6-hourly intervals after insemination until ovulation, which was adjudged by the sudden disappearance of the largest dominant follicle. Measurement of the size of pre-ovulatory follicles was also conducted in both groups. Ultrasound examination was also repeated on days 5 and 12 post AI for measurement of the size of the CL, and on day 30 post AI for pregnancy diagnosis for calculation of the conception rates in both the groups separately [17].

### 2.6. Statistical Analysis

All parameters like plasma LH concentrations, days open, plasma P_4_ concentrations, size of pre-ovulatory follicles and size of CL were analyzed using Student’s *t*-tests. Differences with *p*-values less than 5% (*p* < 0.05) were considered to be statistically significant. All statistical analysis was performed using the SPSS (16.0) system for windows.

## 3. Results

Significantly higher numbers of buffaloes ovulated within 24 h post AI in the control group (82.4%) as compared to only 15.4% in the treatment group. In the treatment group, 53.8% of ovulations occurred over 24 h after AI, whereas in the control group only 11.8% of ovulations occurred over 24 h after AI. Up until 96 h post AI, 30.8% of buffaloes in the treatment group and only 5.9% of buffaloes in the control group remained anovulatory. Pre-ovulatory follicles converted into cysts in all anovulatory buffaloes (Table 1). P_4_ supplementation at the time of insemination was found to delay the time of ovulation and increase the chance of follicular cyst formation in buffaloes if provided during the estrus period.

### 3.1. Levels of Plasma P_4_ at Different Days of Oestrous Cycle

The level of plasma P_4_ at corresponding days of the estrous cycle was found almost similar in treatment and control groups. So, no significant effect of exogenous P_4_ was seen in the form of increased levels of plasma P_4_ on day 5 and day 12 in the treatment group as compared to the control group (Table 2).

### 3.2. Pre-Ovulatory Follicle Size

The ovulatory follicle sizes did not differ between the treatment (15.3 ± 0.7 mm) and control (16.4 ± 0.7 mm) groups. Out of 17 buffaloes of the control group, 16 buffaloes ovulated and one buffalo developed a follicular cyst, whereas out of 13 buffaloes in the treatment group, nine buffaloes ovulated and four developed follicular cysts (Table 3).

### 3.3. Size of CL on Day 5 and Day 12 of the Estrous Cycle

**Figure 1:**
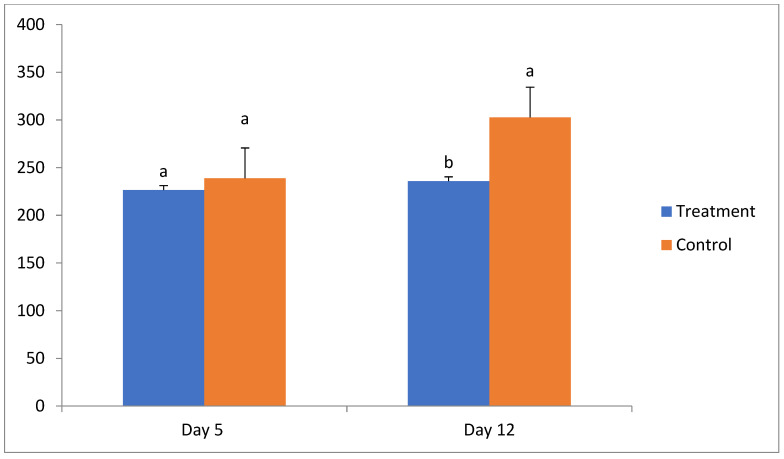
Size of corpus luteum (CL area in mm^2^) on days 5 and 12 of the estrous cycle in treatment and control groups. a, b—different superscripts indicate significant differences (*p* < 0.05).

The corpus luteum area (CLA) was calculated using a formula (CLA = CL length × 0.5 × CL width × 0.5 × 3.14) as described earlier [18]. No significant difference was found in CL size on day 5 of the estrous cycle between the treatment (226.5 ± 17.4 mm^2^) and control (238.9 ± 7.9 mm^2^) groups. However, on day 12 of the cycle the size of the CL was significantly less in the treatment group (235.8 ± 33.5 mm^2^) compared to the control group (302.6 ± 12.4 mm^2^) (Table 4, Figure 1).

### 3.4. Conception Rate

Following insemination, 52.9% of buffaloes in the control group conceived, whereas in the treatment group only 38.5% of buffaloes conceived (Table 5). A tendency of delayed ovulation was observed in treatment group buffaloes. Interestingly, one buffalo in the treatment group that ovulated between 42 and 48 h of insemination also conceived. Of the nine buffaloes in the control group that conceived, seven had ovulated within 24 h and two buffaloes ovulated after 24 h. On the other, of the five buffaloes in the treatment group that conceived, two had ovulated within 24 h and three after 24 h.

## 4. Discussion

P_4_ plays an important role in the regulation of the estrous cycle and pregnancy maintenance. The dynamic pattern of peripheral P_4_ release is similar in cattle and buffaloes during the estrous cycle [1,2,3]. The concentration of P_4_ is minimal on the first day of the estrus cycle (0.1 ng/mL), rises to peak concentrations of 1.6–3.6 ng/mL on days 13 to 17 of the cycle, before declining to basal levels at the onset of next estrus cycle [4]. Administration of hydroxyprogesterone after insemination did not result in significant variation in the level of P_4_ on days 0, 5 and 12 of the estrous cycle (0.5 ± 0.1; 1.1 ± 0.1; 1.5 ± 0.2 ng/mL), as compared to animals in the control group (0.5 ± 0.0; 1.3 ± 0.1; 1.8 ± 0.1 ng/mL) on their respective days of cycle. Regressing CLs were not detected on ultrasound scanning at the time of AI in any members of either group.

It was observed in our study that ovulation was delayed in buffaloes that were injected with P_4_ at the time of insemination. In the control group, 82.4% of buffaloes ovulated within 24 h post AI, whereas in the treatment group only 15.4% of buffaloes ovulated within this period. Furthermore, in the treatment group, 53.8% of animals ovulated within 24 h of AI, whereas in the control group only 11.8% of ovulations occurred within 24 h of insemination. It was found that 30.8% of buffaloes in the treatment group remained anovulatory until 96 h after insemination, whereas in the control group only 5.9% buffaloes did not ovulate. The preovulatory follicle converted into a cyst in all these anovulatory buffaloes. These findings clearly indicate that exogenous P_4_ injections at the time of insemination delayed the time of ovulation and increased the chance of cyst formation in buffaloes if administered during the estrus period. The similar levels of P_4_ on days 5 and 12 in both the groups suggests its metabolic degradation in the body. This is supported by [19], who found that there were no significant variations in plasma P_4_ profiles in repeat-breeding dairy cows 7 days after P_4_ injection. The administration of P_4_ had no effect on the estrous cycle length of non- pregnant buffaloes [13]. Others have also suggested that the administration of exogenous hydroxyprogesterone injections to repeat-breeder cows on days 5–7 improves conception rates [20,21]. The administration of P_4_ would support the luteal production of P_4_ and thus probably prevent luteolytic cascade due to embryonic deaths. However, it is sometimes wrongly administered on the first day of the estrus cycle by inseminators who have poor knowledge of these hormones.

Some earlier studies have indicated that elevated P_4_ levels soon after AI result in lower pregnancy rates [5,6,7,8,9]. Others have indicated that minor elevations in P_4_ concentrations near or at the time of AI have detrimental effects on fertility [10,11]. Poor fertility following P_4_ administration can be attributed to the ageing of ovulatory oocytes in delayed ovulators. Cows with a two-wave follicular pattern are less fertile compared to those with a three-wave pattern, as in the two-wave pattern cows’, the oocytes become aged and have low capacity for fertilization and further development [22,23,24,25]. These findings support our results showing that exogenous P_4_ at the time of insemination caused delayed ovulation and anovulation in the P_4_ treatment group, probably due to suppressed LH release. Reduced LH peaks owing to higher levels of P_4_ in the environment also alter sperm or oocyte transport by altering uterine or oviductal contractility and reducing embryo development by reducing endometrial thickness and blastocyst formation rates.

The sizes of pre-ovulatory follicles range from 12 to 18 mm in cattle and buffaloes [26,27]. It has been observed reported that the administration of P_4_ in the early luteal phase did not affect the pre-ovulatory size of follicles, which were comparable in treatment (12.3 ± 0.2 mm) and control (14.2 ± 0.3 mm) groups of Nili-Ravi buffaloes [13]. Similarly, in the present study, no significant difference was seen in pre-ovulatory follicular size in treatment (15.3 ± 0.7 mm) and control (16.4 ± 0.7 mm) groups in Murrah buffaloes. We did not find significant differences in CL area on day 5 of the estrous cycle between treatment and control (226.5 ± 17.4 vs. 238.9 ± 7.9 mm^2^) groups, whereas on day 12 of the cycle, CLA was significantly lower in the treatment group as compared to control (235.8 ± 33.5 vs. 302.6 ± 12.4 mm^2^). Plasma P_4_ assays also showed no higher values in the control group than the treatment group on days 5 and 12 of the estrous cycle. It has been reported that mean corpus luteum areas (CLAs) ranged between 225 and 250 mm^2^ in buffalo heifers [17]. Hence, these findings clearly suggest the negative effects of exogenous progesterone injection at the time of insemination on reproduction parameters. The use of P_4_ at the time of AI is of major concern under field conditions and is not to be advocated for improving conception rates in normal breeders or repeat breeders.

## 5. Conclusions

In conclusion, it was found that P_4_ supplementation at the time of insemination delayed the time of ovulation and increased the chances of follicular cyst formation in buffaloes. Moreover, P_4_ supplementation had no effect on plasma P_4_, ovulatory follicle size or conception rate. Hence, the use of exogenous P_4_ administration at the time of AI has no and/or negative effects on the fertility in buffaloes.

## Figures and Tables

**Table 1:** Ovulatory responses in treatment and control groups.

Group	*n*	Time of Ovulation with Respect to Mid-Heat Insemination								Overall Ovulations		
		0–6 h	6–12 h	12–18 h	18–24 h	24–30 h	30–36 h	36–42 h	42–48 h	<24 h	>24 h	Anovulation
Treatment	13	--	7.7%	--	7.7%	--	7.7%	30.7%	15.4%	15.4% ^b^	53.8% ^b^	30.8% ^b^
Control	17	5.9%	11.8%	11.8%	52.9%	5.9%	5.9%	--	--	82.4% ^a^	11.8% ^a^	5.9% ^a^

a, b—different superscript differs significantly in a column (*p* < 0.05).

**Table 2:** Plasma P_4_ concentrations (ng/mL) in treatment and control groups at different time of estrous cycle.

Groups	*n*	Mean Plasma P_4_ Concentration (ng/mL)		
		At the time of AI	Day 5	Day 12
Treatment	13	0.5 ± 0.1 ^NS^	1.1 ± 0.1 ^NS^	1.5 ± 0.2 ^NS^
Control	17	0.5 ± 0.0 ^NS^	1.3 ± 0.1 ^NS^	1.8 ± 0.1 ^NS^

NS—Non-Significant.

**Table 3:** Pre-ovulatory follicular size (mean ± SE) in treatment and control groups.

Group	*n*	Size of Pre-Ovulatory Follicle (mm)	Animals Ovulated	Follicular Cyst Formation
Treatment	13	15.3 ± 0.7 ^a^	69.2% ^b^	30.8% ^a^
Control	17	16.4 ± 0.7 ^a^	94.1% ^a^	5.9% ^b^

a, b—different superscript differs significantly in a column (*p* < 0.05).

**Table 4:** Size of CL (area in mm^2^; mean ± SE) on day 5 and 12 of the estrous cycle in treatment and control groups.

Group	*n*	CL Formation	Corpus Luteum Area in mm^2^ (Mean + SE)	
			Day 5	Day 12
Treatment	13	9	226.5 ± 17.4 ^a^	235.8 ± 33.5 ^b^
Control	17	16	238.9 ± 7.9 ^a^	302.6 ± 12.4 ^a^

a, b—different superscripts indicate significant differences in a column (*p* < 0.05).

**Table 5:** Animals that conceived (%) with respect to time to ovulation and overall conception rate (CR %) in treatment and control groups.

Group	*n*	Pregnant (*n*)	Animals that Conceived (%) with Respect to Time Interval Between Insemination and Ovulation								Overall CR %
			0–6 h	6–12 h	12–18 h	18–24 h	24–30 h	30–36 h	36–42 h	42–48 h	
Treatment	13	5	--	7.7%	--	7.7%	--	7.7%	7.7%	7.7%	38.5% ^b^
Control	17	9	5.9%	5.9%	5.9%	23.5%	5.9%	5.9%	--	--	52.9% ^a^

a, b—different superscripts indicate significant differences (*p* < 0.05).

## Data Availability

The data supporting the conclusions of this article will be made available by the authors on request.
